# Assessment of the Catalytic Performances of Nanocomposites Materials Based on 13X Zeolite, Calcium Oxide and Metal Zinc Particles in the Residual Biomass Pyrolysis Process

**DOI:** 10.3390/nano12213841

**Published:** 2022-10-31

**Authors:** Elena David, Janez Kopac

**Affiliations:** 1 National Research Institute of Cryogenics & Isotopic Technologies, Street Uzinei No. 4, P.O. Râureni, P.O. Box 7, 240050 Râmnicu Vâlcea, Romania; 2 Faculty of Mechanical Engineering, University of Ljubljana, Askerceva 6, SI-1000 Ljubljana, Slovenia

**Keywords:** nanocomposites, 13X zeolite, CaO, Zn particles, catalyst, residual biomass, pyrolysis

## Abstract

Nanocomposites based on 13X zeolite (13XZ), calcium oxide (CaO) and metal zinc particles (Zn) were prepared. The resulting nanocomposites were characterized by different techniques. Then, a comparative study on catalytic and noncatalytic pyrolysis of biomass waste was performed to establish the influence of nanocomposites used as catalysts on the yields and characteristics of liquid and solid products. Residual rapeseed biomass (RRB) was employed for pyrolysis experiments and a fixed bed reactor was used. By introducing CaO and metal zinc particles into 13X zeolite mass, the surface area (S_BET_) of nanocomposites was reduced, and this decrease is due to the introduction of nano-calcium carbonate and nano-zinc particles, which occupied an important space into zeolite structure. By adding CaO to 13XZ, the pore structure was changed and there was a decrease in the micropores volume. The analysis of the pore area distribution showed a hierarchical pore structure for nanocomposites. The elements composition showed that the main elements contained in nanocomposites are Si, Al, Ca and Zn, confirming the preservation of the zeolite structure. Using these nanocomposites as catalysts in pyrolysis process, the residual biomass could be valorized, producing bio-oil and biochar for the management and sustainability of this low-value waste.

## 1. Introduction

Environmental pollution and degradation (air, water, and soil) is continuously increasing and, to reduce the consequences, the development of new depollution techniques is required [1,2,3]. A cause of the increase in pollution is represented by agricultural wastes that, by decomposing, produce environment pollution when stored on the soil. The recovery of these wastes removes the problems related to their storage, reduces pollution and contamination of the soil and air, and ensures their recycling into useful products [4,5]. For this purpose, valuable products, such as bio-oil, biochar and bio-syngas, can be obtained by thermochemical processing (pyrolysis and gasification) of these biomass wastes [6,7]. One such residual biomass is represented by rapeseed waste, including straw, leaves, pods or rapeseed cake resulting from the extraction of oil from rape seeds, a solid residue rich in organic compounds with a high content of volatile compounds and carbon [8]. The bio-oil produced has applications in the production of fuels [9,10], and biochar can be a good amendment for degraded soils, it offers many advantages, including an improved soil structure and, also, a reduced potential for heavy metal leaching. This is due to its characteristics, such as a relatively large surface area, high pH, high organic C content and the presence of functional groups that can bind to contaminants [11,12,13,14,15]. It can also be a good adsorbent material, as well as a material with catalytic properties [16,17]. In addition to bio-oil and biochar, biomass waste pyrolysis produces a gas fraction (known as bio-syngas), which can be collected and utilized as an alternative to fossil fuels [18].

In recent years, researchers have increased their interest in the valorization of waste from the rape crops and, in this context, new methods for the production of fuels from rapeseed were developed [19], and new methods for renewable energy and biochar production from rapeseed were developed and studied [20]. Rapeseed is one of the most widely planted crops and, after harvesting the rapeseed, the removal of the stalks and pods remains a problem, as well as the residue left after extracting the oil from the seeds; they have no clear utility and returning them to the land is not a sustainable solution. Therefore, the most common elimination, open burning, remained uncontrolled, causing environmental pollution. However, the residual biomass from rapeseed crops represents a particularly promising source of raw material that can be exploited for the production of fuels and biochar [20]. Rapeseed cake, seeds and straw, collected from various areas, have been pyrolyzed by various researchers under different conditions [21,22,23]. In any case, full-scale characterization studies considering the production of fuels and biochar from residual rapeseed biomass are not quite few in the literature.

For example, the biochar was obtained under different pyrolysis temperatures, various heating rates and pyrolysis residence times [21,22], with the aim to establish the relationships with yield of biochar, its pH, and functional groups. Different techniques of investigation were used, such as proximate analysis, elemental analysis, surface area and pores, and surface morphology. These results helped to determine the relationship between the conditions of pyrolysis and the physical and chemical properties of biochar produced from rapeseed residual biomass, and thus supported the establishment of the optimal pyrolysis conditions to maximize the beneficial use and to improve its environmental management. The annual global production of rapeseed is estimated to be several million tonnes; hence, the importance of rapeseed as an energy crop is rising [24]. There are some studies about the investigation of processing of rapeseed by pyrolysis process under various conditions to produce bio-oil that can be processed to fuel [25,26,27]. For example, gross calorific value of bio-oil produced from rapeseed biomass wastes ranges around 18.5–20.5 MJ/kg [26,27] and this makes rapeseed biomass waste a suitable source for bio-oil production for fuel applications.

The thermochemical conversion of biomass includes combustion, gasification, pyrolysis and liquefaction as main processes. Among these, pyrolysis attracted attention to a greater extent because it is able to convert the biomass in various forms of energy, such as biofuel (liquid), biochar (solid) and gaseous products. The carbonization of the materials in the absence or low oxygen content in a range of temperatures of 400 to 700 °C is known as pyrolysis process [28,29]. The products resulting from pyrolysis process can be used as hydrocarbon feedstocks, which represents the primary resource for bio-refineries [30].

The bio-oil resulting from pyrolysis presents several disadvantages, such as lower pH value (increased acidity), high viscosity, lower heating capacity and a higher oxygen content, which decrease the bio-oil stability during storage and reduce the flame temperature when it is used as fuel for engines [31,32]. These disadvantages can be removed by improving fuel quality using different methods, such as catalytic cracking and deoxygenation [33,34]. To achieve this goal, new types of catalysts must be developed. Together with other catalysts, nanocomposites based on zeolites and metal oxides could be effective in such applications. Hari Prasad Reddy Kannapu et al. [33] studied pyrolysis of sawdust for production of bio-jet fuels using activated carbon catalysts with various contents of MgO. The catalysts have a high potential in the conversion of oxygenated compounds to the jet fuel. They concluded that the nanosized particles of MgO and the acid and base sites of catalysts enhanced the deoxygenation reactions by decarboxylation and decarbonylation, which are required for fuel formation in high amounts from waste biomass pyrolysis. Jilai Wu et al. [34] studied the fast pyrolysis of alkali lignin in black liquor in a fixed bed reactor to prepare bio-chemicals. The effect of NaOH catalyst on the preparation of aromatic hydrocarbons and the effect of pyrolysis temperature and the catalyst-to-lignin ratio on the yield and relative content of aromatic hydrocarbons were investigated. The results showed that the addition of NaOH increased the in situ H_2_ supply and improved the removal of methoxyl groups; thus, the content of aromatic hydrocarbons increased and oxygen content decreased. Putun Ersan [35] studied the pyrolysis of cotton seeds at 550 °C, with and without catalysts, and the obtained results demonstrated that the use of catalysts reduced the yield of bio-oil but improved its quality regarding the calorific value, removal of oxygenated compounds and changed the hydrocarbon distribution. Moreover, the metal oxide nanocatalysts, such as Al_2_O_3_ and MgO, act on the active site of biomass, such as biomass pore, and improve the physical properties, so it is presented in a recent study [36]. In addition, the use of metal nanocatalysts assures a higher catalytic efficiency due to low co-ordinated sites, which increase the reaction rate and reduce the catalyst amount used [37].

The use of zeolite having nanoparticles in framework structure significantly increases the aromatic hydrocarbon content and decreases the oxygenated compounds from bio-oil composition [34]. It was also observed that the use of zeolite catalyst decreased the liquid yield and increased the yield of char and gas [38]. The analysis of the literature in the field highlights that few studies are reported and refer to the use of nanocomposites based on zeolite in the pyrolysis of residual rapeseed biomass (RRB) for the production of renewable fuel (bio-oil) and char with properties improved. With these findings, and according to the authors’ knowledge, the catalytic pyrolysis of RRB using cheap catalysts has not been studied extensively and in detail.

Thus, this experimental study focused on the synthesis and characterization of nanocomposites based on zeolite 13X, calcium oxide and metal zinc particles, the use of these nanomaterials in pyrolysis process and the analysis of the effect of operating parameters, such as temperature, heating rate, biomass particles size, carrier gas flow rate (N_2_), as well as the impact of the nanocatalyst loading degree on the yield and characteristics of bio-oil produced. Further, based on the physical and chemical characteristics, the liquid (bio-oil) and solid (biochar) products were characterized by different techniques, such as elemental analysis (CHNS), thermogravimetric analysis (TGA), X-ray diffraction (XRD), gross caloric value (GCV), gas chromatography–mass spectroscopy analysis (GC–MS), scanning electron microscope (SEM) and Fourier-transform infrared spectroscopy (FTIR), and water holding capacity (WHC). CaO was added to zeolite to help the cracking of heavy compounds into several light oxygenated compounds; then, the modified zeolite 13X was used for converting light compounds to aromatic compounds. Combining CaO, metal zinc particles and zeolite 13X resulted in nanocomposites with good catalytic properties and it was proved that their use as catalysts could be a potential way to improve the quality of bio-oil during pyrolysis process [9].

## 2. Materials and Methods

### 2.1. Residual Rapeseed Biomass

**Figure 1:**
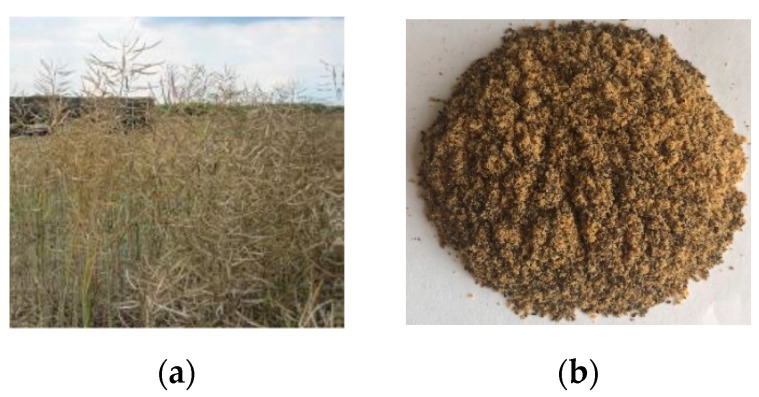
Residual rapeseed biomass: (**a**) gross residual rapeseed biomass (straw, leaves, and pods); (**b**) residual rapeseed biomass having particle size of 0.5 mm.

In this study, residual rapeseed biomass (RRB) that included straw, leaves and pods was used, and this was collected from the local area (Figure 1a,b).

The RRB was dried at 105 °C for 6 h and, then, was shredded and sieved and stored in airtight glass containers to avoid the absorption of humidity. The resulting biomass was labeled as residual rapeseed biomass (RRB). The moisture, fixed carbon, volatile and ash content, the ultimate and proximate analysis were determined according to standards ASTM D 2016 74, D3174 89, D1102 84 and ASTM 128 D 5373 using a Carlo Elba 1106 instrument. The RRB composition was determined according to ASTM D 3176 and the ASTM D240-02 standard was employed to determine the gross calorific value (GCV). The extractives and lignocellulosic content of RRB was established using TAPPI method, as is presented in the reference [39]. The proximate and ultimate analysis results are presented in Table 1. The organic structure of RRB was established by Fourier infrared spectroscopy and an FTIR spectrometer Nicolet 6700 was used. The dried sample was blended with KBr and the resulting mixture was placed in the sample holder. The scanning was performed at the rate of 40 with a step size of 4 cm^−1^ and in a wavelength range of 400–3900 cm^−1^. Moreover, the bio-oil FTIR analysis was made using attenuated total reflectance (ATR). A small drop of bio-oil was placed on the ATR crystal surface and the analysis was made with the procedure described above. The thermal stability of RRB was determined by thermal analysis using a TGA analyzer type Netzsch, TG-209 F1 Libra in a N_2_ gas atmosphere. About 10.0 ± 0.2 mg of solid sample was heated from room temperature to 900 °C at 10 °C/min heating rate, with a N_2_ flow rate of 50 mL/min.

### 2.2. Nanocomposites Preparation

The materials used were of analytical grade and were purchased from Sigma-Aldrich (Burlington, MA, USA). The 13X zeolite was in pellets form (Ø = 3 mm), with a Si/Al ratio = 3.2, a specific surface area of ~370 m^2^/g and the pore volume of 0.25 cm^3^/g. The 13X zeolite was provided by Fluka of Sigma-Aldrich Holding AG. The initial 13X zeolite was milled and sieved and the zeolite particles with a size of 180–220 nm were obtained. The main precursor materials used to prepare the nanocomposites were 13X zeolite and nano-CaCO_3._ The methods to prepare three kinds of nanocomposites (hereafter called catalysts) are the following: (1) 1#13XZ catalyst. The 500 g of untreated 13X zeolite was treated in an electric furnace for 3 h at 180 °C and, then, the temperature furnace was increased to 550 °C and 13X zeolite was calcined at this temperature for 4 h. This sample was labeled as 1#13XZ_T_; (2) 2#CaO/13XZ catalyst. The nano-CaCO_3_ (≤150 nm) was calcined at 800 °C in air for 30 min to obtain the CaO. A total of 28 g CaO was put into the 2500 mL of distilled water under continuous stirring to obtain a 0.2 M calcium hydroxide solution, Ca(OH)_2_. A total of 300 g of 1#13XZ was added into the alkaline solution and, then, the solution was stirred constantly at 85 °C for 2 h (a suspension solution resulted). A total of 172 g nano-CaCO_3_ was put in the above solution under constant stirring until a colloidal solid was obtained. The product was dried in an oven at 105 °C until complete drying and, then, the powder was calcinated at 550 °C for 2 h; (3) 3# CaO-Zn/13XZ catalyst. An amount of Zn(NO_3_)_2_•6H_2_O (0.05 mol) and 172 g nano-CaCO_3_ were added and dissolved in 200 mL of suspension solution as it was prepared in method 2. After that, an appropriate volume of distilled water was added into the suspension until the mixed solution reached a volume of 1000 mL. The other operation and treatments were the same as 2#CaO-13XZ catalyst.

### 2.3. Nanocomposites Characterization

The phases and crystallinity of the nanocomposites (catalysts) were established by analysis of X-ray diffraction (XRD), using D/max-2200/PC, Rigaku, Japan, and copper KR radiation (40 kV, 20 mA) as the X-ray source. The analysis was made at room temperature in the range of 2θ from 5° to 85°, with a scanning rate of 2°/min. The N_2_ adsorption/desorption isotherms of the catalysts at (−196.15 °C) were measured using Quantachrome Inst., Nova 2200e analyzer, and the Brunauer–Emmett–Teller (BET) method was used to determine the surface area, the pore volume distribution at a relative pressure of P/P_o_ = 0.99 and the pore size distribution resulting from the Brunauer–Joyner–Halenda (BJH) model. The elements composition of fresh nanocomposites was determined by using the method of energy-dispersive X-ray fluorescence (EDXRF). Scanning electron microscopy (SEM) was used to analyze the nanocomposites morphology (SEM) and a microscope JSM-7500 F (JOEL-Japan, Tokyo, Japan), operated at 10 kV, was used, using gold coating. The TPD-NH_3_ experiment was performed to analyze the acid-based characteristics of the fresh nanocomposites (catalysts). A TPD Nuchrom analyzer with a thermal conductivity detector was used to perform the TPD-NH_3_ experiment. Moreover, the TPD CO_2_ experiment was used to investigate the adsorption characteristics of the nanocomposites (catalysts) used in the pyrolysis process. For each experiment, 0.2 g of sample was used and the procedure was as follows. The sample was pretreated for 30 min at 250 °C and, after, the sample was cooled to room temperature, using He as carrier gas at a flow rate of 40 mL/min. Then, the sample was absorbed by NH_3_ or CO_2_ gas until saturation level; this step lasted for 10 min. After that, the sample was heated according to the conditions of temperature programmed, such as heating rate at 10 °C/min and the He gas flow rate of 40 mL/min. Both NH_3_-TPD and CO_2_-TPD experiments were performed starting from room temperature to 800 °C.

### 2.4. Residual Rapeseed Biomass Pyrolysis Experiments Using Nanocomposites as Catalysts

The yield of pyrolysis products is influenced by some parameters, such as temperature, residence time, heating rate, flow rate of the carrier gas, particle size, kind of biomass, types of the reactor and feedstocks, type of pyrolysis process (with or without catalyst), etc. In this study, pyrolysis temperature, the heating rate, the size of biomass particles and the flow rate of carrier gas were varied to obtain maximum pyrolysis liquid yield. The pyrolysis test was carried out for different temperatures, such as 450, 500, 550, 600 and 650 °C, different heating rates of 25, 50, 75, 100 and 110 °C/min, different particle sizes of 0.5, 1, 1.5, 2 and 2.5 mm and different gas flow rates of 40, 60, 80, 100 and 120 mL/min.

**Figure 2:**
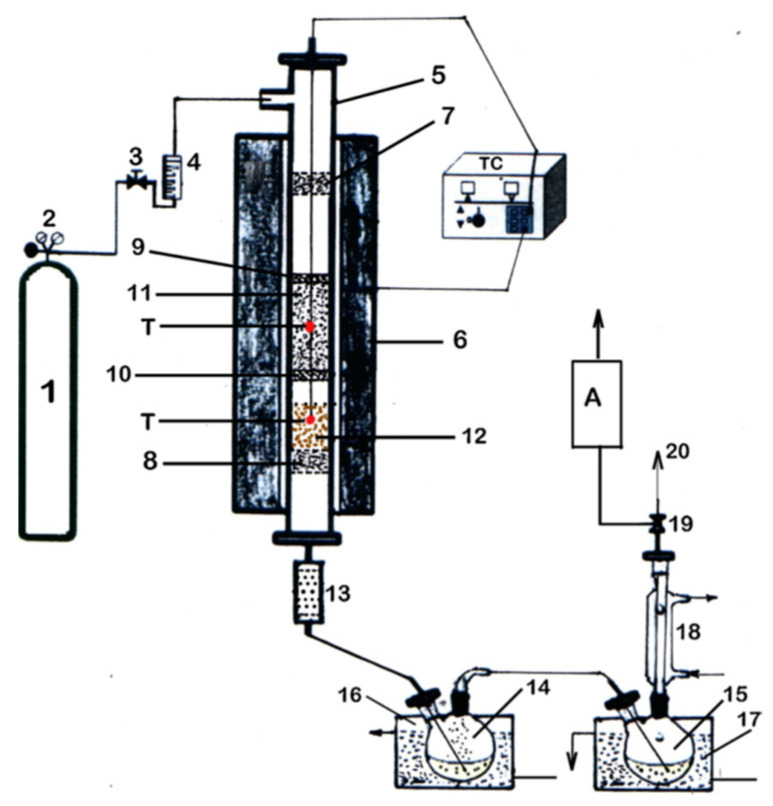
Scheme of experimental setup for biomass pyrolysis. (1)—N_2_ tank; (2)—pressure controller; (3)—adjusting valve; (4)—flowmeter; (5)—reactor; (6)—electrical furnace; (T)—temperature control; (7) and (8)—metallic sieve; (9) and (10)—metallic perforated support; (11)—biomass bed; (12)—catalyst bed; (13)—ceramic filter; (14) and (15)—liquid product collector; (16)—cooling bath (85 °C); (17)—cooling bath (10 °C); (18)—condenser with water (5 °C); (19)—two-way valve; (20)—gas product. (A)—analyzer.

The experimental test includes two stages: pyrolysis stage and catalyst regeneration stage. The experiments of thermal and catalytic pyrolysis were carried out under N_2_ gas in a fixed bed reactor (tubular reactor made of stainless steel, having a length of 700 mm and inner diameter of 12 mm) heated by electrical furnace. The schematic diagram is shown in Figure 2.

The temperature was measured in the middle of the biomass and catalyst bed using Cr/Al thermocouples. The N_2_ flow rate was set by a fine control using a gas flowmeter. For every pyrolysis experiment, 50 g of residual rapeseed biomass was used and, to investigate the catalyst effect on pyrolysis product yields and bio-oil characteristics, various catalyst loading (10, 20 and 25 wt.%, respectively) were used, which represented a ratio biomass to catalyst of 10, 5 and 4, respectively. The upper layer is residual rapeseed biomass and the lower layer is catalyst (Figure 2). Before each pyrolysis experiment, the whole system is stripped with N_2_ gas to remove the air and to assure inert atmosphere during the experiment. During thermal pyrolysis, the catalyst was not loaded in the reactor. During catalytic pyrolysis, both biomass bed and catalyst bed were loaded in the reactor. The obtained thermal or catalytic pyrolysis vapors were next passed through a ceramic filter to retain any particles of solid and, then, the liquid was collected by cold traps. The solid products consisting of bio-char and catalyst with coke deposited on and into its mass were weighed to establish the pyrolysis yields. Each pyrolysis experiment was repeated at least three times, and the error of measurement was less than 0.5%. Point values on the diagrams represent an average value of these data.

### 2.5. Products Characterization

The product yields (solid, liquid and gas) were determined using the following equations:

% Y_Char_ = W_Char_/W_Total feed_ × 100 (1)

% Y_Liquid_ = W_Liquid_/W_Total feed_ × 100 (2)

% Y_Gas_ = 100 − (% Y_Liquid_ + % Y_Char_) (3)

where % Y_Char_ represents yield of char; % Y_Liquid_ represents yield of liquid; % Y_Gas_ represents yield of gas; and W_Total feed_ represents the weight of total feed.

The liquid produced consists of a bio-oil fraction and an aqueous fraction. From this mixture, the bio-oil fraction was separated from aqueous fraction by extraction method using diethyl ether as solvent. For this, the liquid product was mixed at a ratio of 1:1 with diethyl ether. The separated bio-oil fraction was dried over anhydrous calcium chloride, and then evaporated in a rotary evaporator at 25 °C in order to remove extraction solvent (diethyl ether) [40,41]. The organic fraction was weighed, bottled and labeled as bio-oil product.

The resulting bio-oil was analyzed using GCMS-QP2010SE Gas Chromatograph/Mass Spectrometer. The temperature of GC/MS injector was kept at 270 °C and the temperature of interface was 300 °C. The chemical compounds were separated on a capillary column having the following dimensions: 30 m × 0.25 mm × 0.25 μm, using helium as carrier gas at a flow of 0.8 mL/min and the mass spectrometer was used at 75 ev with a scan rate of 30–550 amu per second. The typical compounds were identified using the NIST database of MS spectra [42]. The peak area (%) and the relative content of compounds were determined by area normalization and were used to estimate the products’ composition. The analysis of the functional groups was performed with a Nicolet 6700 FTIR spectrophotometer using tablets of KBr of 1 mm thickness. The density of bio-oil was determined according to standard ASTM D 1298. The content of moisture in bio-oil was determined using Karl Fischer method and according to standard ASTM D 1744. The content of ash was determined according to standard ASTM D 482 and the content of sulfur according to the IP336 method and ASTM D 4294. The residue of carbon was determined according to standard ASTM D 189, the gross caloric value (GCV) according to standard ASTM D 3286-91a and total acid value according to standard ASTM D 974. The pH of bio-oil was established with a pH meter (Mettler Delta 340), empirical formula and H/C, N/C and O/C molar ratios were calculated based on elemental composition. The bio-oil viscosity was measured by an ARES TA rheometer and the temperature was set at 25 °C and 60 °C.

The released gas product was collected in the gas bags every 6 min, and then analyzed by a chromatograph Varian CP-3800 having TCD and FID detectors and equipped with a 5 Å molecular sieve column of 2 m length and a diameter of 0.00315 mm, a Porapak N column of 6 m length and a diameter of 0.00317 and a Chromosorb column of 1 m length and a diameter of 0.0018 mm. Argon was used as carrier gas and the CO, CO_2_, H_2_, N_2_, CH_4_ and hydrocarbons up to C_7_ concentrations were determined.

The biochar characteristics were established by the proximate and elemental analysis, by acidity, gross heating value and bulk density determination, as in Section 2.1. Moreover, BET surface area and water holding capacity were determined. The BET surface area, pore volume and the surface morphology of the biochar were determined as in Section 2.3. The acidity (pH) of residual rapeseed biochar (RRC) was determined with a pH meter (Mettler Delta 340). Moreover, the capacity of water holding (WHC) of RRC was established and, for this, a ceramic Buchner funnel, Fisher filter paper, size P8, and deionized water were used. A known amount of RRC, free of moisture, was put in the ceramic Buchner funnel having attached filter paper, and a known volume of deionized water was gradually passed on the sample until the biochar was saturated and allowed for water elimination for nearly 4 h. The capacity of water holding (WHC) was obtained by the difference between initial amount of deionized water used and the amount of eliminated water.

## 3. Results and Discussion

### 3.1. Residual Rapeseed Biomass Characterization

Residual rape biomass (straw, leaves and pods, Figure 1) provided by farmers from the local area was characterized and the data are presented in Table 1. To limit the moisture content that could be reached in the bio-oil product, the moisture amount in biomass should be below 10 wt.% [43,44].

As can be seen in Table 1, the moisture content in residual rapeseed biomass was 8.41 wt.%, under 10 wt.% value. The GCV was 19.47 (MJ/kg) and is similar to other residual biomass, such as sunflower stalks [45].

The presence of higher carbon content (42.65%) and lower N_2_ content (4.12%) was confirmed by ultimate analysis, and the lower content of N_2_ could lead to a lower NOx formation during pyrolysis. Moreover, the chemical analysis of RRB confirmed a content of 46.96% cellulose, 16.71% hemicellulose and 27.35% lignin. Moreover, the extraction study showed a percent of 8.98 wt.% of extractives in RRB.

**Figure 3:**
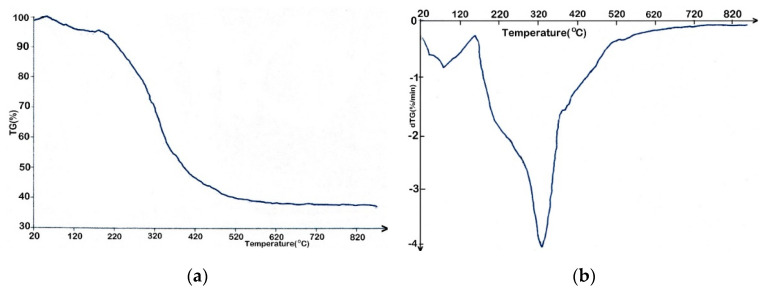
Thermal stability of RRB at 10 °C/min heating rate. TG curve of RRB (**a**) and DTG curve of RRB (**b**).

Thermal analysis of RRB was performed under N_2_ atmosphere and non-isothermal conditions. The diagrams are shown in Figure 3. The thermal profile of RRB decomposition showed that biomass passed through three main stages: first stage of drying (up to 160 °C), the second stage of active pyrolysis in the temperature range 160 to 550 °C and the third stage that is passive or the stage of char formation at a temperature over 550 °C (Figure 3a).

Based on the thermal profile of RRB decomposition, it was deduced that 6.03 wt.% mass loss took place due to moisture removal by dehydration reactions and the evaporation of lighter volatile compounds.

Moreover, in the second stage, about 70.57 wt.% of mass was lost and was due to decomposition reactions of main constituents from biomass composition (cellulose and hemicellulose) that determined the release of a mixture of condensable and non-condensable gases. Moreover, in this stage, compounds with big molecules were fragmented in smaller molecules due to the continuous heat supply. In the third stage, the lignin decomposition took place at a temperature over 550 °C but, due to the presence of hydroxy phenolic group, the process took place at a slower rate that could result in the char formation [46]. The DTG analysis (Figure 3b), confirmed that moisture removal and light volatile compounds took place up to 160 °C and, in the second stage, hemicellulose and cellulose decomposed in light molecular compounds. In the third stage, the lignin decomposition took place over 550 °C, with a slow rate, and it did not result in any sharp peak.

**Figure 4:**
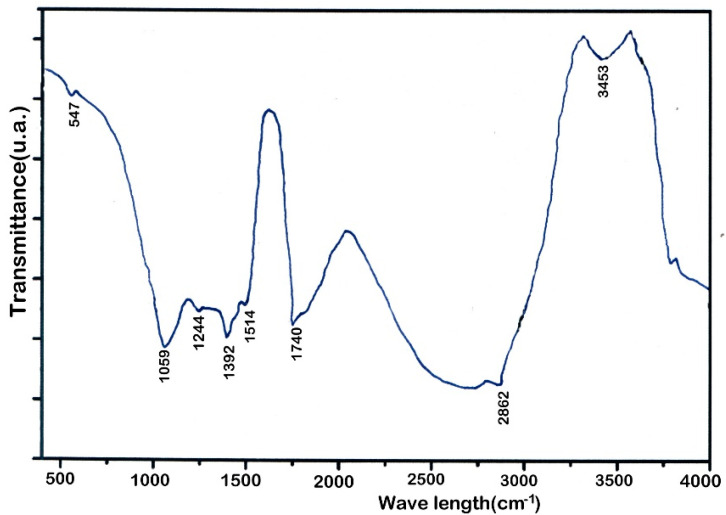
Functional groups of RRB determined by FTIR analysis.

The FTIR spectra of the residual rapeseed biomass is shown in Figure 4. The band of adsorption over 3000 cm^−1^ (3453 cm^−1^) appeared due to the existence of −OH group, which proved the presence of compounds such as acids, phenols, aromatics, as well as water impurities [47]. Furthermore, the band of adsorption at 2862 cm^−1^ was due to the C–H vibration and shows the existence of olefin [48]. The peak at 1740 cm^−1^ was due to C=O stretching vibration and attests to the presence of compounds such as ketones and esters [49]. The peak appearing at 1514 cm^−1^ was due to C=C stretching vibration and confirmed the presence of hydrocarbons (alkene and aromatics) [48,49], while the peak at 1392 cm^−1^ was due to C≡C stretching vibration and showed the alkyne presence.

The peak at 1244 cm^−1^ was due to C–O group and attested to the ether and ester presence [49]. Moreover, the peak at 1059 cm^−1^ was due to C–O vibration and confirmed the acids presence [47] and the peak at 547 cm^−1^ confirmed the presence of mono- and polycyclic aromatic compounds.

### 3.2. Nanocomposites Characterization

From N_2_ adsorption/desorption isotherms, BET surface area (S_BET_) and total pore volume (V_T_) were calculated (Table 2).

The surface area of 1# 13XZ nanocomposite was 371.21 m^2^/g, which was a little bigger than that of the raw 13X zeolite (nontreated 13XZ). The S_BET_ of 2# CaO/13XZ and 3# CaO-Zn/13XZ nanocomposites was 187.65 m^2^/g and 167.48 m^2^/g, respectively. It is clearly seen that the S_BET_ of modified 13X zeolite decreased due to the introduction of nano-calcium carbonate and nano-zinc particle, which occupied an important space in the zeolite structure.

**Figure 5:**
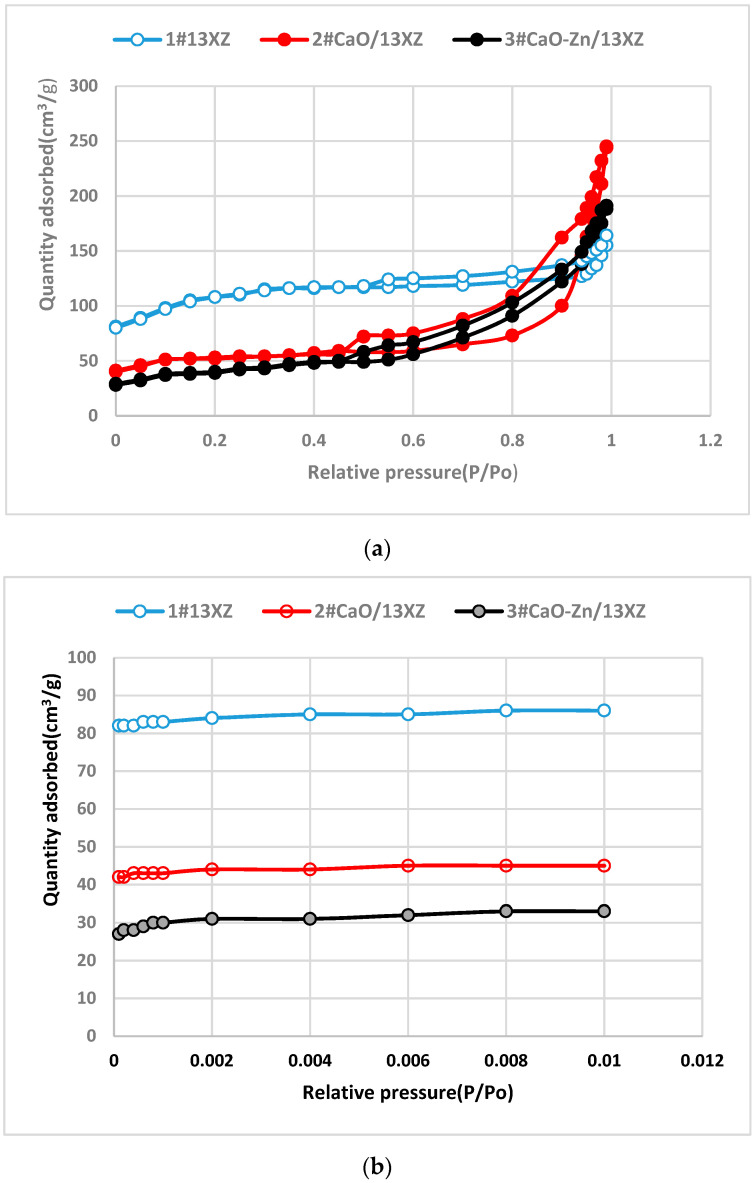
N_2_ adsorption/desorption isotherms for 1# 13XZ, 2# CaO/13XZ and 3# CaO-Zn/13XZ nanocomposites; (**a**) adsorption/desorption isotherms at relative pressure P/P_o_ in the range 0 to 0.99; (**b**) adsorption isotherms at relative pressure P/P_o_ in range 0 to 0.01.

**Figure 6:**
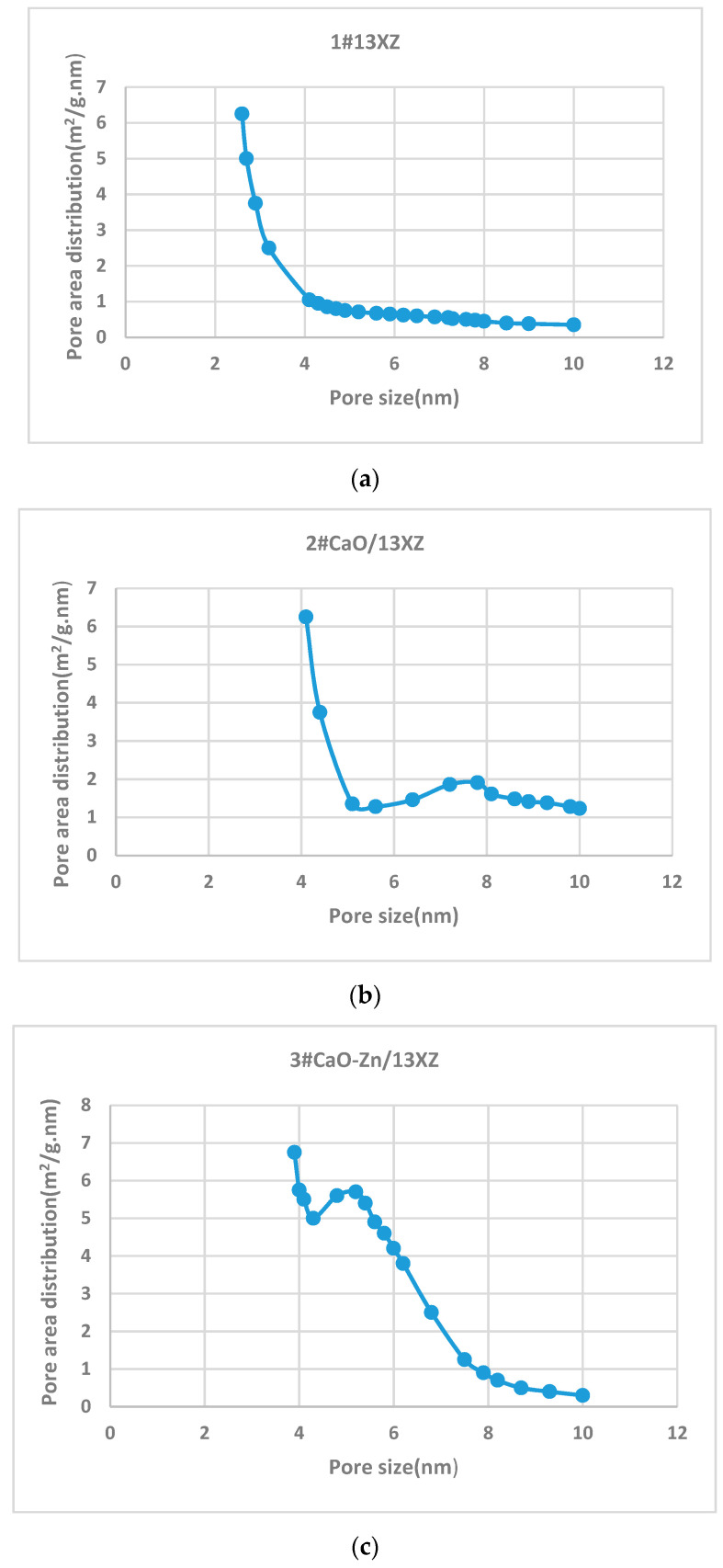
Pore area distributions of: (**a**) 1# 13XZ; (**b**) CaO/13XZ; (**c**) CaO-Zn/13XZ.

The N_2_ adsorption/desorption isotherms of 1# 13XZ, 2# CaO-13XZ and 3# CaO-Zn/13XZ nanomaterials are presented in Figure 5. As can be observed the N_2_ quantity adsorbed on 2# CaO-13XZ and 3# CaO-Zn/13XZ, nanocomposites presented the same tendency at a relatively low pressure but they had a lower value than that of 1# 13XZ. By adding CaO to 13XZ, the pore structure was changed and N_2_ absorbed volume decreased, showing a decrease in the volume of micropores. Moreover, the initial zeolite (1# 13XZ) and nanocomposites 2# CaO-13XZ and 3# CaO-Zn/13XZ exhibited adsorption of gas (N_2_) below the pressure value of P/PO = 0.01, which confirms the presence of micropores in the structure of the materials (Figure 5b). On the other hand, the increase in the amount of gas adsorbed at a relative pressure of more than 0.9 is due to the presence of intracrystalline mesopores. The occurrence of hysteresis during desorption showed the presence of mesopores for nanomaterials samples 1#, 2# and 3# (Figure 5). Most of the pores in the 13X zeolite structure are <50 nm in size; therefore, the macropore volume is extremely small. On the other hand, as can be seen in Figure 6a–c, the predominant distribution of mesopores is in the range below 10 nm.

The occurrence of hysteresis during desorption showed the presence of mesopores for nanomaterial samples 1#, 2# and 3# (Figure 5). The analysis of the pore area distribution (Figure 6) for modified 13X zeolite showed a hierarchical pore structure mainly in 2# CaO/13XZ and 3# CaO-Zn/13XZ nanocomposites, which are in agreement with the results shown in Figure 5. The porosity of the samples varies and this is due to the fact that the micropores and mesopores with smaller sizes (<4nm) initially present in the 13X zeolite, by adding CaO and Zn, are filled with these particles and, thus, most of the mesopores are located in the range >4 nm.

**Figure 7:**
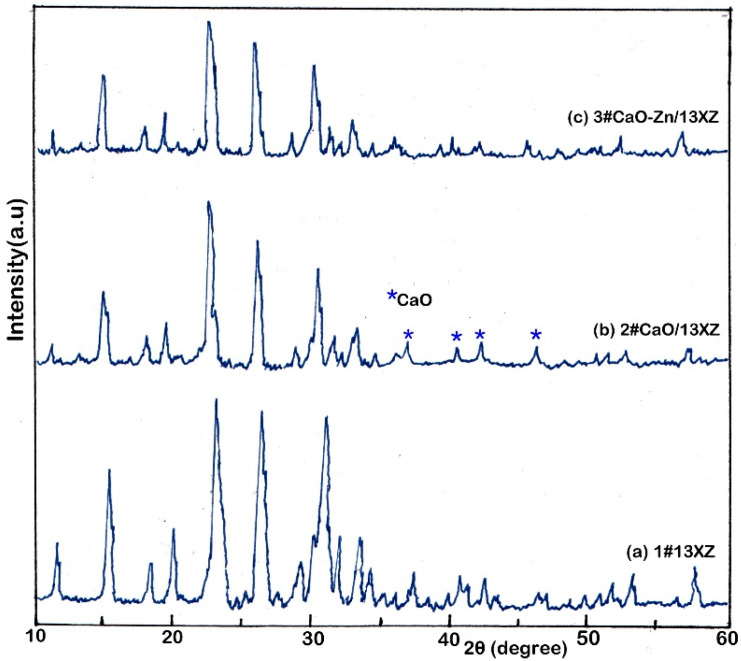
XRD spectra of: 1# 13XZ nanomaterial (**a**), 2# CaO/13XZ nanocomposite (**b**) and 3# CaO-Zn/13XZ nanocomposite (**c**); * CaO-the peak confirms the presence of CaO in zeolite mass.

The XRD spectra of the nanocomposites are presented in Figure 7, where the spectra (a) represent the 1# 13XZ nanomaterial. It can be seen that the spectrum shows characteristic peaks of faujasite-type structure [50].

For the 2# and 3# nanocomposites, most parts of the original peak (13XZ) of the nanomaterials remained unchanged, while CaO was found in the 2# CaO/13XZ spectrum as characteristic peak. The elements composition of the nanocomposite samples was established by energy-dispersive X-ray fluorescence (EDXRF) and the results are presented in Table 3. It can be observed that the samples contain as main elements Si, Al, Ca and Zn and these are basic components of nanocomposites.

**Figure 8:**
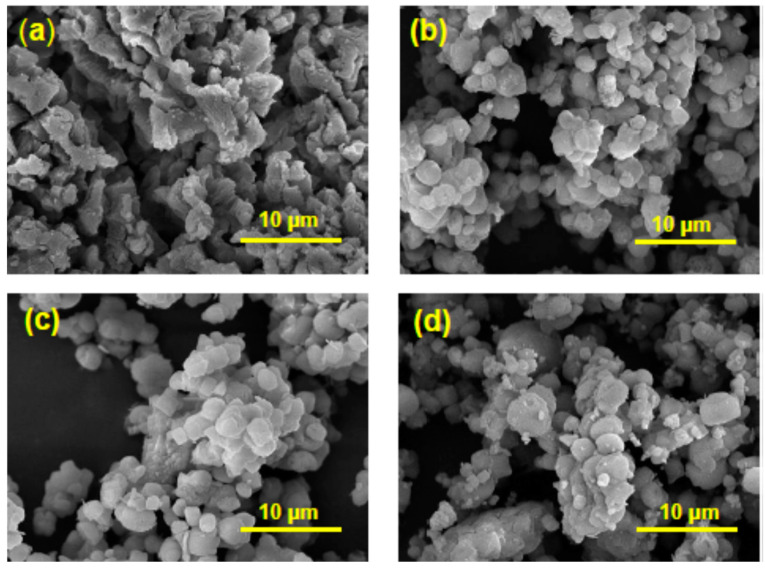
SEM images of (**a**) CaO, (**b**) 13XZ, (**c**) CaO/13XZ and (**d**) CaO-Zn/13XZ.

The added metal Zn was found in nanocomposite 3# CaO-Zn/13XZ in a percentage of 11.53%. The SEM images of CaO and 13X zeolite, as well as of 2# CaO/13XZ and 3# CaO-Zn/13XZ nanocomposites are shown in Figure 8. As can be seen from Figure 8a, the CaO oxide contains particles of irregular shape with different sizes that are connected between them and show as a nest-like structure. On the other hand, the 13X zeolite (Figure 8b) appears with a regular shape containing smaller aggregates and shows the presence of pores. Moreover, CaO/13XZ sample (Figure 8c) presents a morphology similar to 13XZ, showing that the 13X zeolite network was preserved even after the CaO was loaded into the zeolite mass. At the same time, it can be seen from Figure 8d. that the surface of nanocomposite 3# CaO-Zn/13XZ is rougher, suggesting a change in the surface morphology, probably due to the presence of metallic zinc particles.

**Figure 9:**
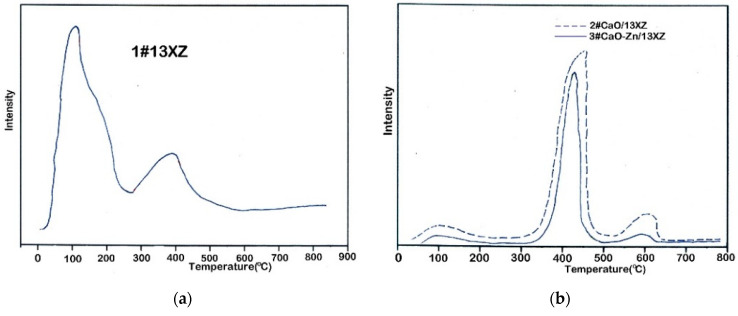

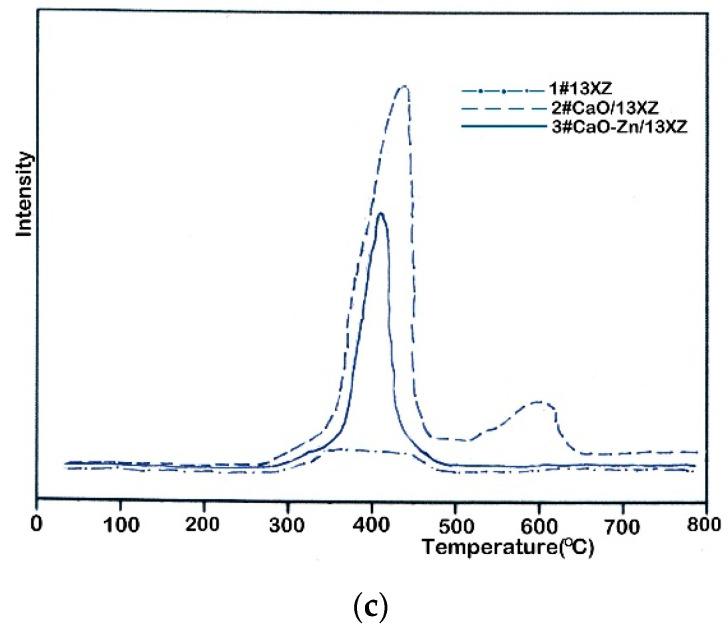
NH_3_-TPD diagrams: (**a**) NH_3_-TPD diagram of fresh 1# 13XZ; (**b**) NH_3_-TPD diagrams of fresh 2# CaO/13XZ and 3# CaO-Zn/13XZ nanocomposites; (**c**) CO_2_-TPD results of the used 1# 13XZ, 2# CaO/13XZ and 3# CaO-Zn/13XZ.

The NH_3_-TPD curves of the 1# 13XZ, 2# CaO/13XZ and 3# CaO-Zn/13XZ fresh materials are shown in Figure 9a,b.

Figure 9a presents the NH_3_-TPD diagram of 1# 13XZ and Figure 9b shows the NH_3_-TPD diagrams of 2# CaO/13XZ and 3# CaO-Zn/13XZ nanocomposites, respectively. The 1# 13XZ fresh material showed three peaks of desorption that can be associated with the presence of acid sites, weak sites for the peak that appeared at temperature range of 105 to 120 °C, sites with medium acidity for the peak appearing at the range 185 to 210 °C), and sites with strong acidity for the peak appearing at 390 to 410 °C, respectively. After the addition of CaO and impregnation of Zn(NO)_3_.6H2O, the sites with weak acidity remained largely unchanged but the corresponding peak substantially decreased.

On the other hand, it can be observed that the intensity of the peak corresponding to strongly acidic sites increased substantially after the addition of CaO as well as Zn. For 2# CaO/13XZ and 3# CaO-Zn/13XZ nanocomposites, it appeared there was another strong acid site corresponding to 580–620 °C. In 2# CaO/13XZ and 3# CaO-Zn/13XZ catalysts, the acidity increased, which could be due to the creation of new sites with acidic character by introducing of CaO and Zn on the support.

The dispersion of CaO and Zn particles on support modifies the acid character of catalysts by reducing the number of Brönsted sites but increasing the number of Lewis sites. According to these findings, the increase in total acidity resulted from the increase in the number of Lewis acid sites [31,32]. The decrease in the number of Brönsted acid sites may be related to the addition of CaO and, also, of the impregnation process with the metal precursor when maybe some hydrogen ions (H+) present in the zeolitic support were exchanged by Ca or Zn ions.

The CO_2_-TPD analysis was performed to evaluate the performances of the nanocomposites used as catalysts in the pyrolysis process. From Figure 9c, it can be observed that the used 1# 13XZ did not respond to the CO_2_-TPD test and, with the temperature increasing, no peak of significant intensity appeared. However, 2# CaO/13XZ and 3# CaO-Zn/13XZ catalysts showed clear intensity peaks in the temperature range 390–410 °C and 580–620 °C, respectively. These could be attributed to the decomposition of CaCO_3_ that was probably formed during the pyrolysis process by combining the resulting CO_2_ with the CaO present in the mass of the catalysts, according to the reaction (4):

CaO + CO_2_ ↔ CaCO_3_ (4)

It can be concluded that 2# CaO/13XZ and 3# CaO-Zn/13XZ catalysts could be used to reduce the amount of CO_2_ released during the pyrolysis process and to improve the composition of bio-syngas produced.

### 3.3. The Analysis of Process Parameters

**Figure 10:**
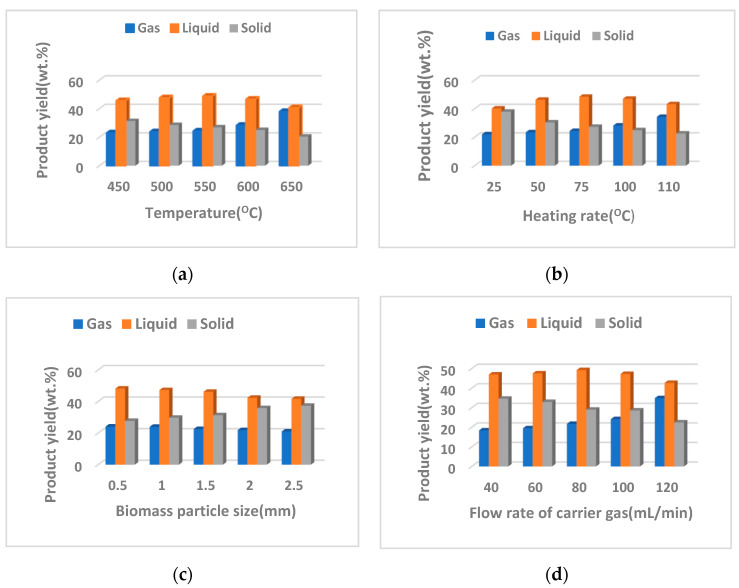
The product yield depending on the variation of the parameters of the pyrolysis process.

The effect of pyrolysis process parameters (temperature, heating rate, biomass particle size and carrier gas flow rate) on the product yield is shown in Figure 10a–d.

The obtained results showed that a maximum liquid yield of 48.73 wt.% resulted at a pyrolysis temperature of 550 °C, which indicates that, at this temperature, the pyrolysis process was complete and led to the formation of a larger amount of condensable volatile compounds. On the other hand, it was observed that, at a lower pyrolysis temperature (450 or 500°C), the char yield was higher (31.29 wt.% and 28.55 wt.% for 450 °C and 500 °C, respectively), while the gas yield changed very little (23.06 and 23.81 wt.% for 450 °C and 500 °C, respectively), but the liquid fraction increased from 45.64 to 47.63 wt.% for a pyrolysis temperature of 450 °C and 500 °C, respectively. These results indicate that, at temperatures of 450 or 500 °C, the biomass pyrolysis process is partial and, consequently, a higher yield of solid (char) is produced.

At the higher pyrolysis temperature of 600 and 650 °C, respectively, the gas yield increased (28.32 wt.% for 600 °C and 38.01 wt.% for 650 °C, respectively), while the liquid and solid yield decreased (Figure 10a). These results are due to a rapid process of decomposition of biomass components into non-condensable volatile gaseous compounds.

The influence of heating rate on product yield is shown in Figure 10b. It was noticed that, for a heating rate of 75 °C/min, a maximum liquid yield of 48.37 wt.% was obtained, while the char yield was 27.26 wt.% and the gas yield was 24.35 wt.%, respectively, much lower. These results are due to a maximum transfer of mass and heat between the particles that led to a complete pyrolysis of the biomass and to a maximum of volatile compounds released. On the other hand, it was found that, at lower heating rates, such as 25 or 50 °C/min, the char yield was higher, while the liquid and gas fraction was reduced, these results being due to a lower transfer of mass and heat between biomass particles and an incomplete biomass pyrolysis. Further, at high heating rates, such as 100 or 110 °C/min, the gas yield increased significantly (28.14 wt.% for 100 °C/min or 34.26 wt.% for 110 °C/min, respectively), while the liquid and solid yields decreased, which proves that, at high heating rates, a fast endothermic process of decomposition of biomass into non-condensable gases takes place, these results being in agreement with other studies [21,35].

The effect of biomass particle size on the product yield is shown in Figure 10c. It can be observed that the maximum for bio-oil yield (48.16 wt.%) was obtained for 0.5 mm biomass particle size, while the char and gas yields were lower (27.71 and 24.12 wt.%, respectively) and this is due to a high mass and heat transfer between the particles of biomass taking place; the small biomass particle size has a bigger surface area, which determines a maximum mass and heat transfer between particles and much more volatiles are formed that are condensable and transform in the bio-oil. Moreover, the small biomass particles were rapid and uniformly heated due to high heat transfer. On the other hand, it was observed that, at a bigger particle size, such as 1.5, 2 and 2.5 mm, the bio-oil and gas yields decreased, whereas the char yield increased and these results are due to an incomplete pyrolysis because the larger biomass particles have a lower surface area and, as a consequence, a reduced heat transfer takes place and a formation of a smaller amount of condensable and non-condensable volatiles.

The influence of the carrier gas flow on the product yield is presented in Figure 10d. The maximum bio-oil yield of 49.24 wt.% resulted for a 80 mL/min gas flow rate, whereas the gas and char yields at the same gas flow rate were of 21.74 and 29.01 wt.%, respectively. At a lower gas flow rate (i.e., 40 mL/min), a higher char yield of 34.61 wt.% was obtained and gas and bio-oil yields decreased (18.43 wt.% and 46.95 wt.%, respectively). The increased char yield was due probably to the secondary reactions that took place and which led to the formation of more char. On other hand, at higher gas flow rates of 100 or 120 mL/min, the liquid yield (47.26 wt.% and 42.67 wt.%) and char yield (28.57 wt.% and 22.47 wt.%) decreased, while the yield of gas increased (24.16 wt.% and 34.85 wt.%). This behavior is probably due to the increase in the transport speed of the formed vapors that did not have enough time to decompose into condensable products and, thus, the liquid fraction decreased. All the results presented in this work are in agreement with other studies in the field [21,35].

Analyzing the results obtained, in terms of the effects of temperature, heating rate, biomass particle size as well as carrier gas flow, it can be concluded that a temperature of 550 °C, heating rate of 75 °C/min, 0.5 mm biomass particle size and a carrier gas flow rate of 80 mL/min may be the optimal parameters for the pyrolysis process.

### 3.4. Influence of Nanocomposites Used as Catalysts on Product Yield

**Figure 11:**
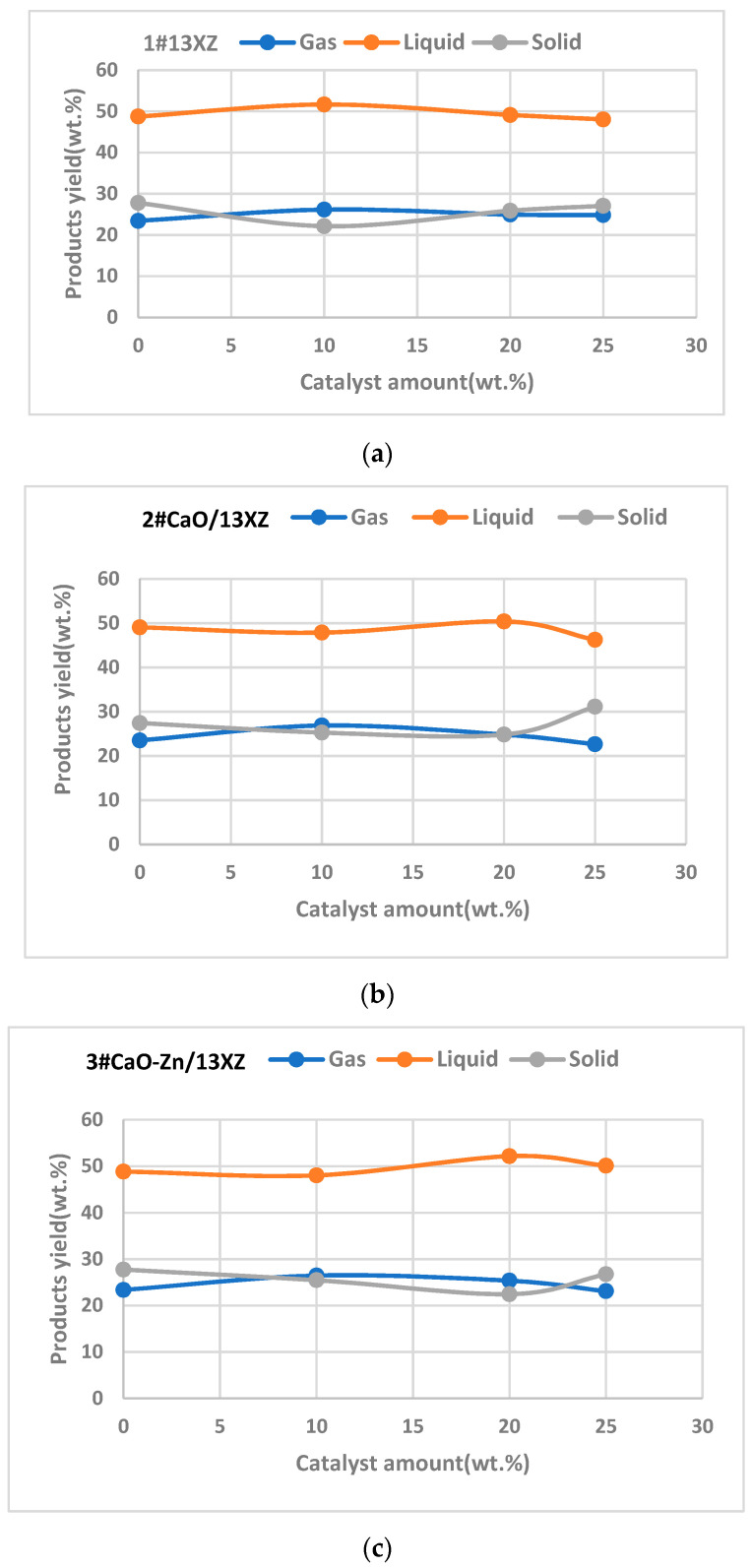
The influence of nanocomposites used as catalyst on product yields; (**a**)—using 1# 13XZ catalyst; (**b**)—using 2# CaO/13XZ catalyst; (**c**)—using 3# CaO-Zn/13XZ catalyst.

The influence of catalysts on the product yield is shown in Figure 11a–c.

A biomass/catalyst (B/C) ratio of 5:1 (respectively, the introduction of a quantity of 20% catalyst) produced a bio-oil yield of 49.14 ± 1.12, 50.37 ± 1.15 and 52.16± 1.20 wt.% for 1# 13XZ, 2# CaO/13XZ and 3# CaO-Zn/13XZ, respectively, compared to an average amount of 48.88 ± 1.20 wt.% obtained without catalyst. This increase is due to the interaction between the biomass and the catalyst, which favored the decarboxylation and decarbonylation reactions and more volatiles were condensed into liquid [51]. From Figure 11, it can be seen that, at a loading of 10 wt.% catalyst, the bio-oil yield was smaller due to lower interaction between biomass particles and catalysts, except for 1# 13XZ catalyst. In the case of 1# 13XZ, a loading of 10 wt.% catalyst probably ensured that pyrolysis vapors enter in the pores of the 13X zeolite, which were not blocked by the presence of CaO and Zn, instead of being mostly adsorbed on the external surface of the catalyst, as probably happened in the case of catalysts 2# and 3#. Moreover, at a lower catalyst loading, the decarboxylation and decarbonylation reactions did not take place significantly, which determined a lower bio-oil yield, as was also reported in the study by Anand et al. [52].

Moreover, it was found that, at higher loading of catalyst (25 wt.%), the bio-oil yield decreased, while the char yield increased, compared with a catalyst loading of 20 wt.%.

This could be due to the coke that resulted during higher loading of catalysts that contributed to the increasing of the char yield. In these conditions, the biomass pores probably were blocked due to a higher loading of catalyst, which determined a higher resistance between biomass and catalyst and the interaction was weaker; thus, the bio-oil yield decreased.

### 3.5. Bio-Oil Product Characterization

The physical and chemical characteristics of the bio-oil resulting from thermal and catalytic pyrolysis process are shown in Table 4 and these are compared with the characteristics of diesel fuel.

Analyzing the obtained results, it was observed that the use of nanocatalysts increased the carbon amount (76.45, 76.04 and 76.71 wt.% for 1#, 2# and 3# nanocatalysts, respectively) compared with bio-oil obtained without catalyst (62.12 wt.%). Biomass pyrolysis in the presence of nanocatalysts reduced the oxygen content from bio-oil composition (12.08, 11.75 and 11.07 wt.% for 1#,2# and 3# nanocatalysts, respectively) compared with 26.81 wt.% without catalyst. This reduction was due to the reaction between oxygen and hydrogen molecules with the formation of water, which further passed into the gaseous phase. The presence in the bio-oil of high content of oxygenated compounds decreased energy density, gross caloric value (GCV) and increased the fuel viscosity. It was observed that the use of nanocatalysts reduced the bio-oil viscosity (as can be seen in Table 4); this could be explained by the fact that the molecules of oxygen reacted with the hydrogen and the resulting water reduced the bio-oil viscosity by decreasing the oxygenated compounds from bio-oil. As can be seen from the data presented in Table 4, although the use of catalysts in the pyrolysis process reduced the viscosity of the bio-oil, it still has a high value compared to that of diesel, which indicates that further treatment is needed substantially to obtain a bio-oil with characteristics similar to diesel. Moreover, the results obtained in the presence of catalysts show that the moisture content increased compared to the bio-oil obtained in the absence of catalysts, which proves that the presence of catalysts favored dehydration reactions, findings presented in other studies [20,54]. Moreover, the use of nanocatalysts led to obtaining bio-oil with increased GCV and lower acidity compared with the bio-oil produced without catalyst use (Table 4). Our results are in agreement with bio-oil characteristics presented in other studies [20,27,54].

**Figure 12:**
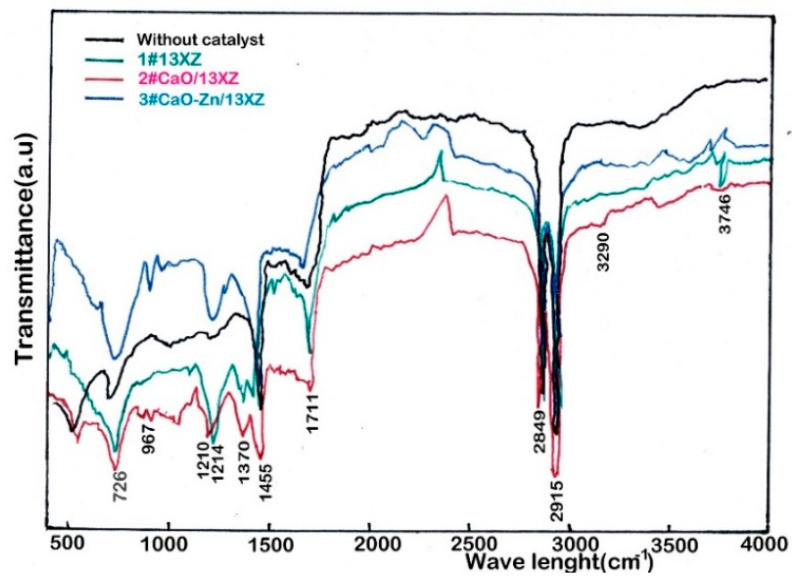
The functional groups determined by the FTIR analysis for catalytic and noncatalytic pyrolysis bio-oil.

The FTIR spectra for bio-oil produced by biomass pyrolysis without and with nanocatalysts are shown in Figure 12. The peaks appearing in the range 680 to 750 cm^−1^ indicated the presence of mono- and polyaromatic compounds. The peaks in the range 950 to 1300 cm^−1^ are related to CO stretching vibration and indicated the presence of alcohols. The peaks appearing in the range 1370 to 1460 cm^−1^ (1455 cm^−1^) were associated with −CH vibration, indicating the presence of a methyl group. The peaks at 1711 cm^−1^ corresponding to C=O vibration indicated the presence of aldehydes, ketones and esters. Furthermore, the peaks in the range 2800 to 3000 cm^−1^ (2849 cm^−1^ and 2916 cm^−1^) correspond with C–H stretching (C–H_3_ and C–H_2_) and showed the presence in the bio-oil of saturated aliphatic groups. On the other hand, the adsorption band in the range 3290 to 3746 cm^−1^ is attributed to −OH vibration and indicated the presence of water, phenol and aromatic compounds in bio-oil. These results are in good agreement with those presented in other studies [20,22,28].

**Figure 13:**
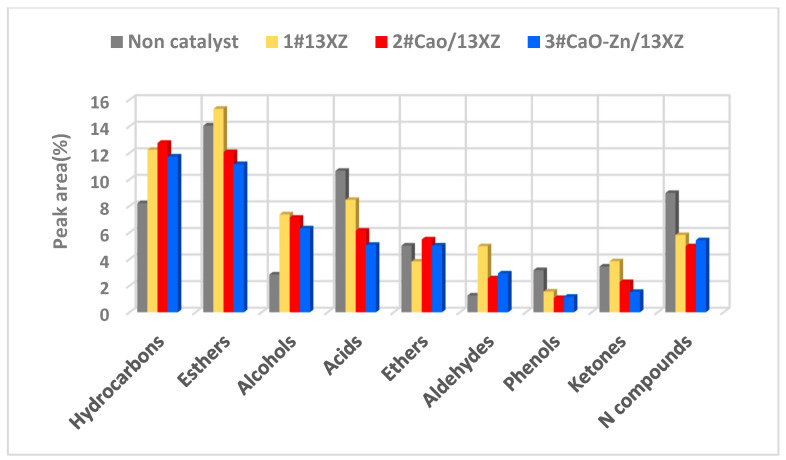
The components determined by GC-MS analysis in bio-oil at composition temperature of 550 °C, heating rate of 75 °C/min, 0.5 mm biomass particle size and a carrier gas flow rate of 80 mL/min.

Through the GC-MS analysis of the bio-oil, the unknown compounds present in the bio-oil were determined and the NIST library was used for identification. For each compound, the specific value at a particular time was determined and the results obtained are shown in Figure 13.

The bio-oil is a complex mixture, containing a lot of compounds, such as aldehydes, ketones, ethers, esters, phenols, acids, alcohols and amide [22,27], and this was confirmed by the GC–MS analysis. By GC–MS analysis of non-catalyst pyrolysis bio-oil, it was determined that there was 8.21% hydrocarbons, 14.05% esters, 4.79% ethers, 2.84% alcohol, 10.65% acids, 5.02% ethers 1.26% aldehyde, 8.98% compound with nitrogen and 3.44% ketones.

These results showed that the bio-oil had in its composition a high fraction of oxygenated compounds and acids that decreased the quality of the bio-oil. Furthermore, the use of catalysts increased the fraction of hydrocarbons (12.23%, 12.75% and 11.72% for 1# 13XZ, 2# CaO/13XZ and 3# CaO-Zn/13XZ, respectively) and this could be due the decarboxylation reaction during pyrolysis in the presence of nanocatalysts, which led to the formation of more hydrocarbons [51,52].

The reactions of decarboxylation employ carboxyl groups and these are removed as carbon dioxide molecules. Moreover, it was observed that the use of catalysts decreased the amount of acids (the area percentage was 8.46%, 6.14% and 5.07% for 1# 13XZ, 2# CaO/13XZ and 3# CaO-Zn/13XZ, respectively) compared with 10.65% for pyrolysis bio-oil obtained without catalysts.

This behavior possibly is due to the fact the nanocatalysts favored the conversion of acids into aldehydes, whose percentage is slightly increased in the presence of nanocatalysts (4.97%, 2.55% and 2.92% for 1# 13XZ, 2# CaO/13XZ and 3# CaO-Zn/13XZ, respectively) compared with 1.26% for pyrolysis bio-oil obtained without catalysts. It could be observed that the area percentage for alcohols was also increased (7.37%, 7.12% and 6.31% for 1# 13XZ, 2# CaO/13XZ and 3# CaO-Zn/13XZ, respectively) compared with 2.84% for pyrolysis bio-oil obtained without catalysts, due to conversion of acids in alcohols [51]. 

The results obtained by GC–MS analysis showed that the use of nanocatalysts decreased the phenols percentage (from 3.17% without catalyst to 1.56%, 1.07% and 1.16% for 1# 13XZ, 2# CaO/13XZ and 3# CaO-Zn/13XZ, respectively), which probably were converted in hydrocarbons. Furthermore, the area percentages of ketones and esters were reduced due to the dehydration reaction during pyrolysis with the formation of water, which contributed to the viscosity reduction (Table 4).

Moreover, the nanocatalyst use decreased the compounds containing nitrogen, probably due the reactions of deamination. These results are in good agreement with other studies [55,56] and, based on them, it can be affirmed that the bio-oil obtained by catalytic pyrolysis could be an alternative to fossil fuels. Among the tested catalysts, the 3# CaO-Zn/13XZ catalyst presented better results regarding bio-oil characteristics (Table 4).

### 3.6. Char Characterization

The solid pyrolysis product obtained at a temperature of 550 °C, heating rate of 75 °C/min, 0.5 mm biomass particle size, a carrier gas flow rate of 80 mL/min, without and using 3# CaO-Zn/13XZ as catalyst was called char from rapeseed waste (RSWC); its characteristics were determined and the results are presented in Table 5.

As can be seen from Table 5, the content of C and H in the RSWC composition is similar to that of the char presented in [16]. The high content in the fixed carbon (46.82%) shows that RSWC could be used for applications such as bio-adsorbent material for separation and purification processes, a source of raw material to produce bio-composites, to produce activated carbons, etc. [8,13,14].

Moreover, it was determined that the organic matter in RSWC was found to be 19.45%, which means that RSWC could be a good material as soil fertilizer. The surface area (S_BET_) of RSWC was determined and is 6.20 m^2^/g, and, as it presented in other studies [16,47], usually this is in the range 1.5 to 500 m^2^/g, which is smaller than the activated carbons (>800 m^2^/g) [9].

The pH of RSWC was determined to be 8.02; this value is in accordance with the results presented in other studies [10,47] and emphasizes that the biochar obtained from biomass pyrolysis is a good improver for the soil [17].

### 3.7. The Stability of 3# CaO-Zn/13XZ Nanocomposite Used as Catalyst

**Figure 14:**
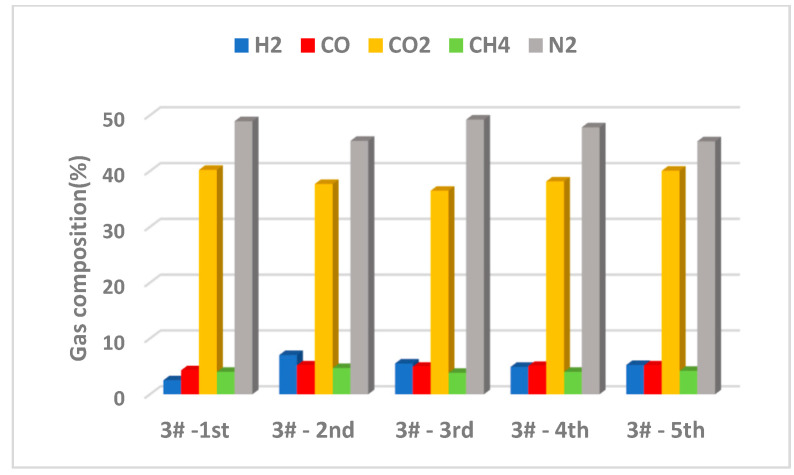
Gas composition for five cycles of experiments using the 3# CaO-Zn/13XZ nanocatalyst.

Considering that the results presented in Section 3.5 showed that the 3# CaO-Zn/13XZ nanocomposite presented better results regarding bio-oil characteristics, when used as a catalyst, the stability of this type of nanomaterial was analyzed (Figure 14).

Five pyrolysis cycles at a temperature of 550 °C, heating rate of 75 °C/min, 0.5 mm biomass particle size and a carrier gas flow rate of 80 mL/min were carried out, with the use of the same catalyst and its transition from one cycle to another. When one cycle test was over, the catalyst was used for a new cycle and, for this, 50 g of fresh biomass was used, 20 g of 3# CaO-Zn/13XZ nanocatalyst and pyrolysis time was 30 min. The evaluation was made according to the composition of the resulting gas after each cycle and the results are presented in Figure 14. During the pyrolysis process, a gaseous fraction containing variable amounts of CO_2_, CO, H_2_, CH_4_ and N_2_ [20,22] is released. CaO from the catalyst composition absorbs a quantity of CO_2_ and transforms into CaCO_3_, as presented in Section 3.2.

It can be observed from Figure 14 that the 3# CaO-Zn/13XZ nanocatalyst presented a difference on gas composition with the number of cycles. For the first cycle, the gas produced contained 4.31% CO and 40.21 CO_2_. It is possible that the amount of CO_2_ was initially higher but a part was absorbed by CaO from the catalyst composition. At the second cycle, the absorption of CO_2_ by CaO probably continued with the further formation of CaCO_3_ and, as a result, the concentration of CO_2_ decreased compared to the first cycle, from 40.21% to 37.68%, respectively. In the third cycle, the CO_2_ amount was still reduced (36.47%), which means that the carbonation process of CaO continued. In the fourth and fifth cycles, the amount of CO_2_ increased again, which would be explained by the completion of the transformation process of CaO into CaCO_3_ after cycle 3 and, in cycle 4 and 5, the CaCO_3_ progressively decomposed, leading to the restoration of CaO and an increase in the amount of CO_2_ due to both the release from CaCO_3_ and the promotion of cracking reactions by CaO.

It can also be observed that the CO_2_ concentration in gaseous product increased gradually, which indicated a lower carbonation reaction of CaO with the cycles’ number and this could also be explained by the produced coke and ash adhering to the catalyst surface, producing deactivation. These results also explain the better behavior of catalyst 3# compared to catalyst 2#, because the zinc content of catalyst 3# maintained a better activity [57,58].

**Figure 15:**
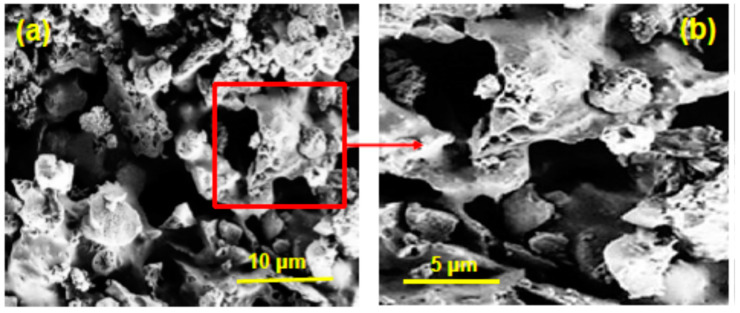
The SEM images for 3# CaO-Zn/13XZ nanocatalyst after the five cycles of pyrolysis: (**a**) Mag = 20.00 KX; (**b**) Mag = 50.00 KX.

Figure 15 shows the SEM images for 3# CaO-Zn/13XZ after the five cycles of pyrolysis and this could be compared with the SEM image for fresh 3# CaO-Zn/13XZ catalyst from Figure 8d.

As can be seen, some particles were partially agglomerated; however, the structure of the specific skeleton of the nanocatalyst was maintained and is similar to the structure of the fresh catalyst, as can be seen from the SEM images (Figure 8d and Figure 15). These findings prove that the 3# CaO-Zn/13XZ nanocatalyst maintains its stability after five pyrolysis cycles.

## 4. Conclusions

The bifunctional CaO/13XZ and CaO-Zn/13XZ nanocomposite materials were prepared and their catalytic performances were evaluated for biomass pyrolysis. The nanocomposites based on 13X zeolite, CaO and metal zinc particles were characterized to investigate their textural characteristics, acidity properties, catalytic performances and the cyclic stability. It was found that the CaO-Zn/13XZ nanocomposite used as nanocatalyst presented both adsorption sites and activated acid sites, which were involved in deoxygenation and cracking reactions for bio-oil production. The surface area of 2# CaO/13XZ and 3# CaO-Zn/13XZ nanocatalysts was smaller than that of the initial 13X zeolite (13XZ). The 1# 13XZ nanomaterial showed three peaks of NH_3_ desorption that were associated with the presence of acid sites (weak, medium and strong acidity sites). After addition of CaO and impregnation of Zn(NO)_3_.6H2O, the sites with weak acidity remained largely unchanged but the corresponding peak substantially decreased. On the other hand, it can be observed that the intensity of the peak corresponding to strongly acidic sites increased substantially after the addition of CaO as well as Zn. The CO_2_-TPD analysis showed that the used 1# 13XZ did not respond to the CO_2_-TPD test and, with the temperature increasing, no peak of significant intensity appeared. However, 2# CaO/13XZ and 3# CaO-Zn/13XZ nanocomposites showed clear intensity peaks in the temperature range 390–410 °C and 580–620 °C, respectively. These could be attributed to the decomposition of CaCO_3_ that was probably formed during the pyrolysis process by combining the resulting CO_2_ with the CaO present in the mass of the nanocatalysts. Further, the effect of these nanocomposites (nanocatalysts) on pyrolysis product yield, as well as the physico-chemical properties of bio-oil, was evaluated. It was observed that the CaO-Zn/13XZ nanocatalyst showed both adsorption and activated acid sites, which were involved in the deoxygenation and cracking reactions to improve the bio-oil quality. Moreover, a part of the amount of CO_2_ released by pyrolysis was captured in the mass of the catalysts due to the CaO content, and a gaseous fraction with a lower CO_2_ content was obtained. These results show that these types of nanocomposites have good characteristics and stability and can be employed in the biomass pyrolysis process to obtain bio-oil with improved quality which can be used as an alternative to fossil fuel.

## Figures and Tables

**Table 1:** Proximate, ultimate and component analysis of rapeseed residual biomass (RRB).

Characteristics	RRB
Proximate analysis (wt.%, db)	
Moisture	8.41 ± 0.11
Volatile matter	70.24 ± 1.35
Fixed carbon	15.53 ± 0.25
Ash content	5.82 ± 0.22
Ultimate analysis (wt.%, db)	
Carbon	42.65 ± 0.21
Hydrogen	5.32 ± 0.25
Nitrogen	4.12 ± 003
Sulfur	0.69 ± 0.01
^a^ Oxygen	47.22 ± 0.25
H/C molar ratio	1.496
O/C molar ratio	0.831
Component analysis (wt.%, db)	
Cellulose	46.96 ± 0.2
Hemicellulose	16.71 ± 0.2
Lignin	27.35 ± 0.2
Alcohol/benzene extractives	8.98 ± 0.2
Empirical formula	CH_1.496_ O_0.831_ N_0.083_ S_0.006_
pH	5.37
^b^ GCVc (MJ/kg)	19.47

db—dry basis; ^a^ calculated from difference; ^b^ gross calorific value; (±) represents the standard error of the mean.

**Table 2:** Surface area and total pore volume of the nanocomposites prepared and used as catalysts.

Nanocomposites (As Catalysts)	S_BET_ (m^2^/g)	V_T_ (cm^3^/g)	Pore Size (A_S_) (nm)
13XZ_NT_ (nontreated)	369.84	0.25	2.58
1# 13XZ_T_ (treated)	371.21	0.24	2.51
2# CaO/13XZ	187.65	0.35	7.41
3# CaO-Zn/13XZ	167.48	0.26	6.34

**Table 3:** The elements composition of the 1# 13XZ, 2# CaO/13XZ and 3# CaO-Zn/13XZ nanomaterials established by EDXRF analysis.

Element (%)	1# 13XZ	2# CaO/13XZ	3# CaO-Zn/13XZ
Si	66.51	43.05	39.42
Al	22.31	7.24	1.25
Ca	1.15	46.35	40.42
P	2.52	1.87	3.11
S	0.31	0.27	0.01
Na	6.74	0.78	4.02
K	0.15	0.12	0.05
Cr	0.03	0.02	0.01
Zn	0.00	0.00	11.53
Fe	0.15	0.16	0.09
Mn	0.005	0.002	0.01
Ti	0.12	0.14	0.07

**Table 4:** Physical and chemical characteristics of the bio-oil obtained from pyrolysis without and with catalyst (temperature of 550 °C, heating rate of 75 °C/min, 0.5 mm biomass particle size and a carrier gas flow rate of 80 mL/min).

Characteristics	No Catalyst	1# 13XZ	2# CaO/13XZ	3# CaO-Zn/13XZ	Diesel [53]
C (%)	62.12	76.45	76.04	76.71	85.63
H (%)	7.68	9.08	9.87	9.95	13.58
^a^ O (%)	26.81	12.08	11.75	11.07	0.66
N (%)	2.51	1.87	1.62	1.55	0.0066
S (%)	0.87	0.51	0.71	0.71	0.11
Density (kg/L) at 25 °C	0.845	0.866	0.862	0.857	0.838
Viscosity (cSt) at 30 °C	68.32	23.19	22.65	22.10	2.12
pH	5.45	5.82	6.85	6.82	-
Moisture (%)	1.95	2.14	2.12	2.38	<0.10
^b^ GHV (MJ/kg)	24.95	32.45	34.65	35.27	45.50

^a^—Calculated from difference; ^b^—gross calorific value; the standard error of the mean was in the range of ±0.01 to ±1.5.

**Table 5:** Physical and chemical characteristics of the char obtained by pyrolysis without catalyst, at a temperature of 550 °C, heating rate of 75 °C/min, 0.5 mm biomass particle size and a carrier gas flow rate of 80 mL/min.

Characteristics	RSWC	Char (Pyrolysis at 500–599 °C) [16]
Moisture (%)	5.07 ± 1.21	-
Volatile matter (%)	35.12 ± 1.45	-
Ash (%)	12.98 ± 0.55	19.2 ± 0.62
Fixed carbon (%)	46.82 ± 1.15	-
Organic matter (LOI) (%)	19.36 ± 0.35	-
C	62.46 ± 1.50	62.3 ± 0.59
H	2.14 ± 0.75	2.83 ± 0.09
^a^ O	31.74 ± 1.75	14.2 ± 0.40
N	3.21 ± 0.21	1.34 ± 0.04
S	0.45 ± 0.15	0.54 ± 0.09
H/C	0.411	-
O/C	0.381	-
Empirical formula	CH_0.411_ O_0.381_ N_0.044_ S_0.003_	-
Density (g/cm^3^)	0.481 ± 1.50	-
Surface area (m^2^/g)	6.28 ± 0.20	97.2 ± 8.48
H_2_O holding capacity (WHC) (%)	40.85 ± 1.12	-
pH	8.02 ± 0.20	9 ± 0.1
^b^ GCV(MJ/kg)	23.45 ± 1.20	-

^a^ calculated from difference; ^b^ gross calorific value; (±) represents the standard error of the mean.

## Data Availability

Not applicable.
